# iTRAQ Proteomic Analysis of Continuously Cropped Soybean Root Inoculated With *Funneliformis mosseae*

**DOI:** 10.3389/fmicb.2019.00061

**Published:** 2019-01-29

**Authors:** Li Bai, Hai-Bing Sun, Rui-Ting Liang, Bai-Yan Cai

**Affiliations:** ^1^Heilongjiang Provincial Key Laboratory of Ecological Restoration and Resource Utilization for Cold Region, College of Life Sciences, Heilongjiang University, Harbin, China; ^2^Department of Food and Environmental Engineering, East University of Heilongjiang, Harbin, China

**Keywords:** continuous soybean cropping, *Funneliformis mosseae*, root rot, iTRAQ proteomic analysis, LC-MS/MS, differentially expressed proteins

## Abstract

Soybean (*Glycine max*) is susceptible to root rot when subjected to continuous cropping, and this disease can seriously diminish the crop yield. Proteomics analyses can show the difference of protein expression in different treatment samples. Herein, isobaric tag for relative and absolute quantitation (iTRAQ) labeling and liquid chromatography-tandem mass spectrometry (LC-MS/MS) were employed for proteomic analysis of continuously cropped soybean inoculated with the arbuscular mycorrhizal fungus (AMF) *Funneliformis mosseae*. The AMF can reduce the incidence of root rot and increase plant height, biomass index in 1, 2, and 4 year of continuous cropping. Differential expression of proteins in soybean roots was determined following 1 year of continuous cropping. A total of 131 differentially expressed proteins (DEPs) were identified in *F. mosseae*-treated samples, of which 49 and 82 were up- and down-regulated, respectively. The DEPs were annotated with 117 gene ontology (GO) terms, with 48 involved in biological processes, 31 linked to molecular functions, and 39 associated with cell components. Kyoto Encyclopedia of Genes and Genomes (KEGG) pathway analysis mapped the DEPs to 113 mainly metabolic pathways including oxidative phosphorylation, glycolysis, and amino acid metabolism. Expression of glucan 1,3-beta-glucosidase, chalcone isomerase, calcium-dependent phospholipid binding and other defense-related proteins was up-regulated by *F. mosseae*, suggesting inoculation promotes the growth and development of soybean and increases disease resistance. The findings provide an experimental basis for further research on the molecular mechanisms of AMF in resolving problems associated with continuous soybean cropping.

## Introduction

Continuous cropping of soybean makes plants susceptible to root rot, resulting in extensive crop damage and economic losses worldwide. The main pathogenic microorganisms causing root rot in soybean include *Fusarium oxysporium* (Lin et al., 2017), *F. graminearum* (Qi et al., 2016), *F. avenaceum* (Wei et al., 2017), *Rhizoctonia solani* (Li et al., 2017) and *Phytophthora. sp* (Puglisi et al., 2017), as well as other fungi. These diseases pose a serious threat soybean yield and quality in China, and morphological and physiological indicators suggest the situation is getting worse.

Arbuscular mycorrhizal fungi (AMF) form specific arbuscular structures and vesicles by infecting the roots of terrestrial vascular plants, thereby enhancing the absorption and transport of carbon, nitrogen, mineral elements and water in plant roots, which promotes plant growth and development, nutritional metabolism, and photosynthesis. AMF are therefore known as biofertilizers (Alva et al., 2017; Luginbuehl et al., 2017), and they have also been associated with resistance to stresses such as salinity and alkalinity, heavy metals, and drought (Tao et al., 2016; Alojz et al., 2017; Shi et al., 2017; Xu et al., 2017), and resistance to diseases including root rot, verticillium wilt and other wilt diseases (Qian et al., 2015; Fauziyah et al., 2017; Sharma and Sharma, 2017; Tripathi et al., 2017). The effects of inoculation with AMF have been widely studied, and the symbiotic relationships between AMF and plants have been analyzed using proteomics. One study revealed differential expression of proteins associated with membrane transport (lipid prions), nutrient uptake and plastid metabolism in the roots of *Medicago sativa* following AMF inoculation (Abdallah et al., 2014). Meanwhile, Bona et al. (2011) found that AMF inoculation induces the expression of stress-related proteins in *Pteris vittata* roots under arsenic stress. For instance, a GTP-binding protein, acetyl-CoA carboxylase, phenylalanine-tRNA ligase and other proteins were significantly up-regulated, thereby reducing the arsenic concentration in parts of *P. vittata* that are below ground (Bona et al., 2011). However, research on the differential expression of proteins in continuously cropped soybean root following inoculation with AMF is minimal.

In the present study, the dominant AMF *Funneliformis mosseae* was inoculated into the soil of continuously cropped soybeans, and protein profiles in soybean roots were analyzed by isobaric tag for relative and absolute quantitation (iTRAQ) labeling and liquid chromatography-tandem mass spectrometry (LC-MS/MS). Differentially expressed proteins (DEPs) following inoculation with *F. mosseae* were subsequently identified, and DEPs were analyzed by bioinformatics to investigate their potential roles in soybean metabolic pathways. The findings provide an experimental basis for research on the molecular mechanism of AMF in resolving problems associated with continuous soybean cropping.

## Materials and Methods

### Ethics Statement

This study uses plants as experimental materials. And the study adhered to other Ethical Guidelines Frontiers in Microbiology.

### Materials

Heinong 48 soybean (high protein type, denoted as HN48; average protein content = 45.23%, average fat content = 18.43%) is planted widely in Heilongjiang Province and was therefore selected as the plant material for these experiments. Soxhlet extraction was used to measure fat content (Wan and Bureau, 2011) and the Kjeldahl method was used to measure protein content (Jung et al., 2003). Experimental soil was collected from the experimental station of the Sugar Institute of Harbin Institute of Technology. The main physical and chemical properties of the soil were as follows: organic matter = 26.14 g/kg, total nitrogen (N) = 1.71 g/kg, total phosphorous (P) = 5.6 g/kg, total potassium (K) 24.9 g/kg, alkaline hydrolysis *N* = 138.1 mg/kg, fast-acting *P* = 13.54 mg/kg, fast-acting *K* = 207 mg/kg, pH = 7.0 (Li et al., 2016).

The *F. mosseae* strain for testing was selected by our research group and deposited at the Institute of Microbiology, Wuhan, China under strain collection number CGMCC No. 3013. *F. mosseae* was propagated using *M. sativa* before planting, and the preparation contained ∼30 spores per g of bacteria.

### Sample Collection and Processing

Experiments were conducted using potted plants, and the production management model was the same as that used on land for growing field crops. Pots of the same size were placed around pots containing soybean plants to protect the line and reduce marginal effects. Treatment group T was inoculated with *F. mosseae*, but Control group C was not. Soil from soybean continuously cropped for 1, 2, and 4 year was used in experiments. A 2 kg sample of air-dried soil was placed in each pot, and 50 g of selected bacterial agent was added and mixed in treatment group pots. Plants were seeded five per pot, and three plants were eventually kept. The experiment included 3 × 2 treatments, with three replicates for C and T groups per treatment.

Treated soybean plants were randomly selected, and debris on the surface of pots was removed. Roots were removed by digging the top 10 - 20 cm soil profile, and fibrous soybean roots were harvested and stored in 10 mL centrifuge tubes. Three biological replicates were included for each treatment, and samples were taken at 60 days after sowing.

### Evaluation of Incidence and Infection Rate

At 60 days after sowing, all root samples from different treatments were randomly selected, and the incidence of disease was counted. All counts were performed in triplicate.

The acid magenta method was used to determine the infection rate. At 30 days after sowing, 50 fibrous roots were randomly selected from each root sample every 10 days, dyed, used to make slides, and subjected to microscopic examination to observe AMF infection in each root segment. The AMF infection rate was counted for each root sample in triplicate.

### Determination of Plant Height and Biomass

Three soybean plants treated in the same way were randomly selected during the growth of soybean, and the height of cotyledon scars to the top of each stem was measured. All counts were performed in triplicate.

In the high-incidence period of soybean root rot, 60 days after sowing, 3 plants were randomly selected in a same treatment. Washed plants with ddH2O and weighed their shoots and roots. Placed shoots and roots in paper bags and water-removing at 105°C for 20 min, then dried until the constant weight at 80°C. Measured biomass.

### Protein Extraction

Protein extraction was performed using sonodynamic therapy (SDT) lysis (Zhu et al., 2014), and the bicinchroninic acid (BCA) method was used for protein quantification. Sodium dodecyl sulfate polyacrylamide gel electrophoresis (SDS-PAGE) was performed to verify protein quality, and protein digestion was conducted by A FFPE-FASP^TM^ Protein Digestion Kit (Protein Discovery, San Diego, CA, United States).

### iTRAQ Markers and String Cation Exchange (SCX) Chromatography

Peptides (100 μg) from each sample were labeled according to the instructions supplied with the iTRAQ Labeling Kit (AB SCIEX, United States) (Applied Biosystems, 2004). Labeled peptides from each group were mixed, and iTRAQ-labeled peptides were fractionated using an AKTA Purifier system (GE, AKTA, Amersham, Pharmacia) equipped with a SCX column.

### LC-MS/MS Analysis

For nanoLC-MS/MS analysis, each peptide mixture was injected onto a reversed-phase trap column (Thermo Scientific Acclaim PepMap100; 100 μm × 2 cm, nanoViper C18) connected to a C18 reversed-phase analytical column (Thermo Scientific Easy Column; 10 cm long, 75 μm inner diameter, 3 μm resin) equilibrated in buffer A (0.1% formic acid) and separated with a linear gradient of buffer B (84% acetonitrile and 0.1% formic acid) at a flow rate of 300 nl/min controlled using IntelliFlow technology. A 1 h gradient was employed as follows: 0-35% buffer B for 50 min, 35-100% buffer B for 5 min, and holding in 100% buffer B for 5 min.

Liquid chromatography-tandem mass spectrometry analysis was performed on a Q Exactive mass spectrometer (Thermo Scientific) coupled to an Easy nLC (Proxeon Biosystems, now Thermo Fisher Scientific) over a 60 min period in positive ion mode. MS data were acquired using the data-dependent top10 method to dynamically choose the most abundant precursor ions from the survey scan (300–1800 m/z) for high-energy collision-induced dissociation (HCD) fragmentation. The automatic gain control (AGC) target was set to 3e6, the maximum injection time was 10 ms, and the dynamic exclusion duration was 40 s. Survey scans were acquired at a resolution of 70,000 at m/z 200, the resolution for HCD spectra was set to 17,500 at m/z 200, and the isolation width was 2 m/z. The normalized collision energy was 30 eV, and the underfill ratio, which specifies the minimum percentage of the target value likely to be reached at maximum fill time, was defined as 0.1%. The instrument was run with peptide recognition mode enabled.

### Statistical Analysis

Raw MS data were analyzed as RAW files. Mascot 2.2 and Proteome Discoverer 1.4 (Thermo Fisher Scientific Inc., 2012) were used for identification and quantitative analysis. For protein quantitation, each protein was required to contain at least two unique peptides. Quantitative protein ratios were weighted and normalized by the median ratio in Mascot^1^. DEPs between *F. mosseae*-treated and control groups were only included if >1.2-fold or <0.833-fold, with *p* < 0.05, and standard proteins were considered differentially expressed.

### Bioinformatics Analysis

Functional classification of DEPs was performed according to gene ontology (GO) annotation and enrichment analysis^2^. DEPs were classified into three categories, namely molecular function, biological process, and cellular component. Kyoto Encyclopedia of Genes and Genomes (KEGG^3^) analysis was used to predict molecular function, biological processes, and pathways associated with DEPs.

Firstly, normalization [to the (-1, 1) interval] of quantitative information for target proteins was conducted by cluster analysis. Secondly, Cluster 3.0 software was used to simultaneously classify the two dimensions of samples and protein expression (distance algorithm, euclid; connection type, average linkage). Finally, a hierarchical clustering heat map was generated using Java Treeview software.

#### Database Selection

Choosing the appropriate protein sequence database is the basis and key step for protein qualitative analysis of mass spectrometry data. This project used the database as: Uniprot_soybean_100396_20160912.fasta (Total number of sequences: 100396, Download time: 2016-09-12, Download link: http://www.uniprot.org).

### Phenylalanine Ammonia-Lyase Activity Assay

At 30 days after sowing, root samples from all treatments were randomly selected every 15 days, and phenylalanine ammonia-lyase activity was determined by borate-mercaptoethanol colorimetry (Gao et al., 2008), in triplicate for each treatment.

### Data Analysis

Data were recorded and initially calculated using Excel. Statistical analysis was performed on the test data using SPSS19 software. The comparison between the means was analyzed by difference significance, *p* < 0.05 was considered significant.

## Results

### Determination of Infection Rate

**FIGURE 1:**
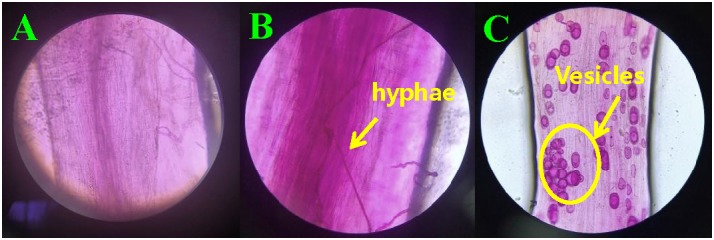
Hyphae and vesicle structures in soybean roots (10 × 40). **(A–C)** infection by *Funneliformis mosseae* in treatment groups at 40, 50, and 60 days after infection, respectively.

At 30 days after planting, the acid fuchsine staining method was used to determine the infection of *F. mosseae* in the roots of continuously cropped soybean every 10 days. As shown Figure 1A Infection by *F. mosseae* was almost not detected in the treatment group at 40 days, and Obvious hyphal structures were observed at 50 days (Figure 1B), and at 60 days after infection, mycorrhizal structures in soybean roots had gradually increased. Hyphae and vesicles were observed by light microscopy (Figure 1C). The AMF infection rate gradually increased with increasing soybean growth and development. At 60 days after infection, the infection rate of *F. mosseae* in the root system of continuously cropped soybean was 100%.

### Determination of Infection Incidence Rate

**FIGURE 2:**
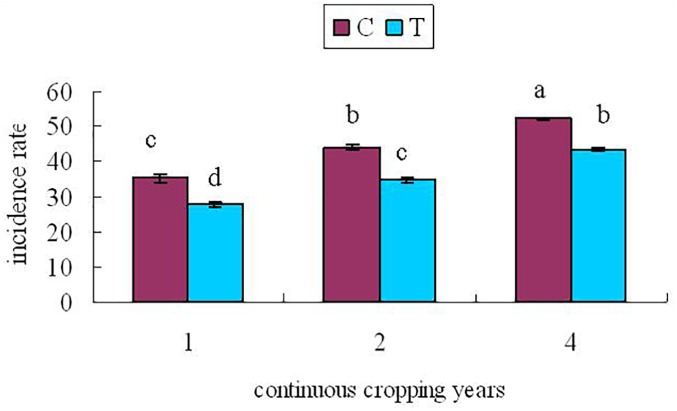
Soybean root rot index At 60 days after planting. C, control group; T, treatment group; x-axis 1, 2, and 4 represents the years of continuous cropping. Vertical bars represent the standard deviation of the means. Lowercase indicate significant differences at 0.05 level.

At 60 days after planting, the roots of continuously cropped soybean were randomly sampled to determine the incidence of root rot in control and treatment groups for 1, 2, and 4 years of continuous cropping. The soybean root rot disease index is shown in Figure 2.

From Figure 2, it can be seen that with the increase of continuous cropping years, the disease index increased significantly (*P* < 0.05). The disease index of the treatment group was lower than that of the control group significantly (*P* < 0.05), indicating that inoculation with *F. mosseae* could alleviate the symptoms of soybean root rot.

### Plant Height and Biomass Index

**FIGURE 3:**
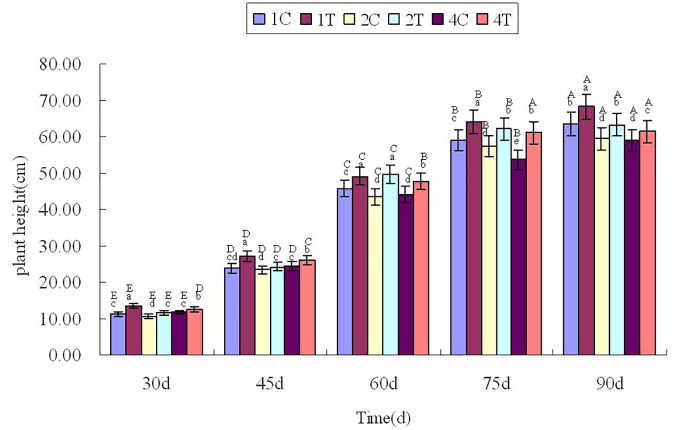
Plant height in continuous cropping soil at different growth stages. C, control group; T, treatment group. 1, 2, and 4 represents the years of continuous cropping. The small letters denote the significant differences among the different treatments at the same growth stages; the capital letters denote the significant differences among the same treatment at different growth stages. The differences in each parameter were detected at *P* < 0.05 level.

Plant height was measured every 15 days after sowing 30 days (Figure 3), Figure 3 showed that the plant height in treatment group were higher than those in control group significantly (*P* < 0.05). It showed that inoculation of *F. mosseae* could alleviate continuous cropping obstacles and promote plant growth. And with the growth of the soybean, plant height increased significantly (*P* < 0.05). The plant height was significantly affected by the sampling time, treatment and their interactions (*P* < 0.05; Table 1).

**Table 1:** Two-way ANOVA for time, treatment and their interactions on plant height, and phenylalanine ammonia-lyase activity.

Index	Item	Time	Treatment	Time × Treatment
Plant height	df	4	5	20
	MS	8777.319	71.487	8.047
	F-ratio	39911.822**^∗∗^**	325.063**^∗∗^**	36.591**^∗∗^**
Phenylalanine ammonia-lyase activity	df	4	5	20
	MS	180.752	104.703	20.075
	F-ratio	472.001**^∗∗^**	273.414**^∗∗^**	52.423**^∗∗^**

*^∗∗^Factors are significant at P < 0.05 level.*

Soybean biomass index was measured at the high incidence period of soybean root rot. Table 2 showed that shoot fresh weight, shoot dry weight and root dry weight in the treatment group were significantly higher than those in the control group after inoculation with *F. mosseae* (*P* < 0.05), It indicated that *F. mosseae* could alleviate continuous cropping obstacles and promote plant growth.

**Table 2:** The biomass index in continuous cropping soil.

Sample	Shoot fresh weight (g)	Shoot dry weight (g)	Root dry weight (g)
1C	63.94 ± 0.5187c	15.45 ± 0.5586c	8.40 ± 0.3001b
1T	103.07 ± 1.3051a	27.20 ± 0.0566a	12.82 ± 0.3656a
2C	48.26 ± 1.2430d	14.69 ± 0.0707cd	6.91 ± 0.2023cd
2T	66.04 ± 0.1159b	20.32 ± 0.2404b	8.77 ± 0.4041b
4C	33.31 ± 1.0883f	8.48 ± 0.2051e	6.07 ± 0.3482d
4T	44.47 ± 1.1229e	14.18 ± 0.4596d	7.47 ± 0.5370c

*Lowercase indicate significant differences at 0.05 level. C, control group; T, treatment group.*

### Protein Quantification

**FIGURE 4:**
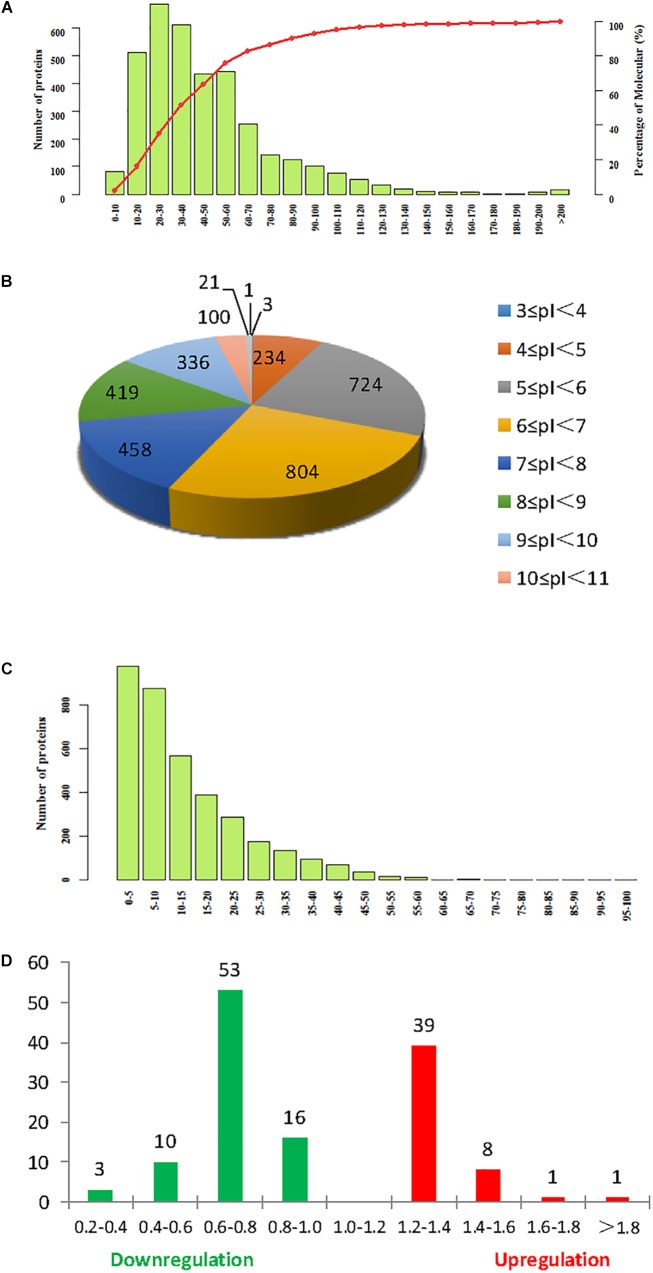
Summary of iTRAQ results. **(A)** Molecular weight. **(B)** Isoelectric point. **(C)** Sequence coverage. **(D)** The degree of up- and down-regulation of differentially expressed proteins (DEPs), colored red, and green, respectively.

Isobaric tag for relative and absolute quantitation labeling was used to identify DEPs in continuously cropped soybean roots inoculated with *F. mosseae*. Comparison with the soybean database revealed a total of 3641 proteins from 11,679 peptides (8836 unique peptides), matching 177,536 spectrograms. Molecular weight, isoelectric point (pI), and peptide sequence coverage distributions are shown in Figure 4A–C, respectively. The molecular weights of most peptides were between 10 and 100 kDa, and only a few were over 200 kDa, while pI values ranged from 5.0 to 9.0, and peptide sequence coverage was >10% for more than half of the identified proteins. And 133 proteins were defined as differentially expressed protein (DEPs) (>1.2-fold or <0.833-fold between *F. mosseae*-treated and control groups, *p* < 0.05). Compared with untreated controls, 49 and 82 proteins were up- and down-regulated in soybean roots following *F. mosseae* inoculation, respectively. Up-regulated proteins were mainly increased between 1.2- and 1.4-fold, while down-regulated proteins were mainly decreased between 0.6- and 0.8-fold. The number of DEPs at different levels is presented in Figure 4D.

**FIGURE 5:**
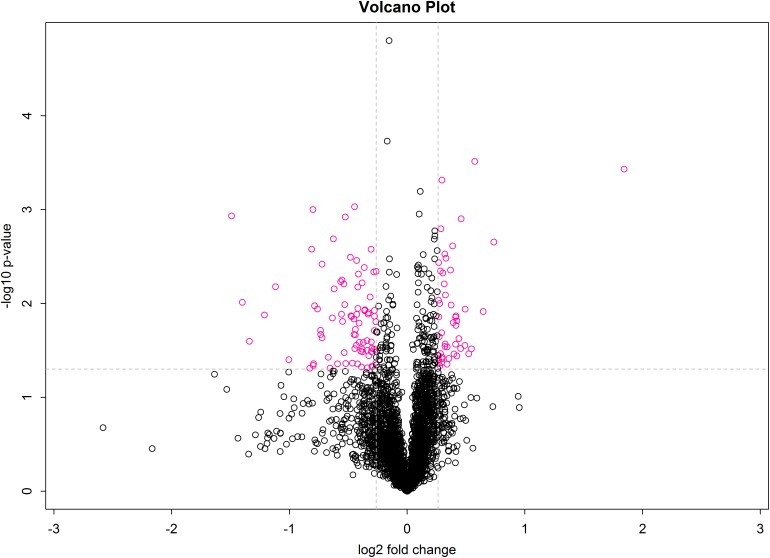
Volcano plot of treatment and control groups. The x-axis represents log2 fold-change values, and the y-axis indicates significant differences in log10 fold-change values.

A volcano plot was drawn using the fold-change in protein expression and the *p*-value obtained from *t*-tests between samples to display significant differences between C and T groups (Figure 5). Differences between T and C groups clearly increase along the x-axis (fold-change) in both directions. Additionally, the larger the value on the y-axis, the more significant the differences between the two groups (red circles represent significant DEPs).

### GO Analysis of DEPs

Gene ontology analysis was performed for functional classification of the identified DEPs, and 2700 GO terms were identified, of which 1551 represented biological processes, 822 were molecular functional processes, and 327 were cellular component processes. A total of 117 GO terms (*p* < 0.05) were detected by Fisher’s exact tests, of which 48 were involved in biological processes, 31 were linked to molecular functions, and 39 were associated with cellular components. These GO terms were classified and enriched, and up- and down-regulated proteins were analyzed.

**FIGURE 6:**
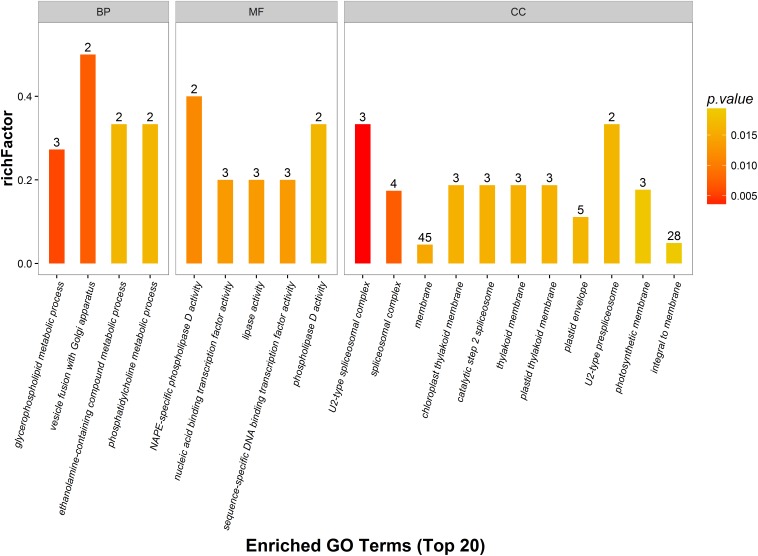
Gene ontology (GO) enrichment analysis of DEPs. DEPs in each group are sorted into three categories; biological process (BP), molecular function (MF), and cellular component (CC). Different GO terms are indicated on the x-axis, and the number of proteins in the indicated categories is indicated on the y-axis.

The distribution of DEPs following inoculation with *F. mosseae* in each GO classification was compared, and the 20 most enriched GO terms and their three main GO categories are presented in Figure 6. Biological processes accounted for four GO terms (three metabolic processes; glycerophospholipids, ethanolamine-containing compounds, and phosphatidylcholine). One cellular process was identified (vesicle fusion with Golgi apparatus), along with five molecular function GO terms (three associated with catalytic activity, and two with binding activity). GO terms enriched in up-regulated proteins were linked to nucleic acid-binding transcription factor activity, sequence-specific DNA-binding transcription factor activity, and phospholipase D activity. GO terms that were enriched in down-regulated proteins were linked to NAPE-specific phospholipase D activity, nucleic acid-binding transcription factor activity, lipase activity, and sequence-specific DNA-binding transcription factor activity. There were 11 GO terms associated with cellular components, and those enriched in up-regulated proteins were membrane, plastid thylakoid membrane, photosynthetic membrane, and integral to membrane. GO terms most enriched in down-regulated proteins were linked to spliceosomal complex assembly, including the aggregation, arrangement and interactions of the ribonucleoprotein spliceosomal complex that catalyzes nuclear mRNA splicing via transesterification reactions.

### KEGG Pathway Analysis of DEPs

**FIGURE 7:**
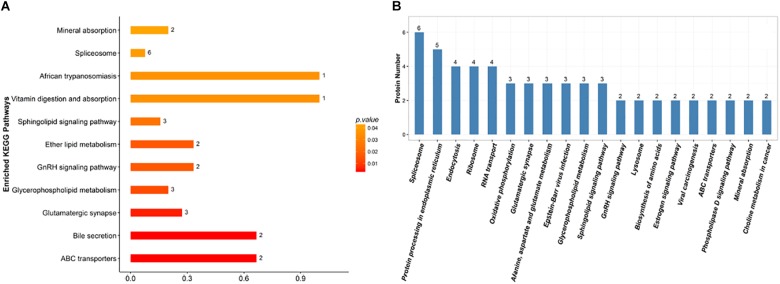
**(A)** KEGG enrichment analysis of the identified DEPs. The x-axis indicates the degree of enrichment (rich factor), and the y-axis represents enriched KEGG pathways. **(B)** Pathways linked to the identified DEPs. The x-axis indicates different KEGG pathways, and the y-axis represents the number of proteins.

Proteins do not usually perform their functions independently, but rather coordinate with each other to perform a series of biochemical reactions. Using established root proteins as the background, significantly enriched pathways were calculated by Fisher’s exact tests, which identified metabolic and signal transduction pathways that were significantly affected by inoculation with *F. mosseae* (Figure 7A). KEGG pathway analysis successfully mapped 131 DEPs to 113 pathways. In the top 20 most enriched pathways following inoculation with *F. mosseae*, a large number of DEPs were associated with oxidative phosphorylation, glycolysis, amino acid metabolism, and glycerophospholipid metabolism. Inoculation with *F. mosseae* also significantly affected mineral absorption, vitamin digestion and absorption, for which many DEPs were up-regulated. The absorption of minerals and vitamins is consistent with the observed strong growth of the treatment group. Furthermore, up-regulation of oxidative phosphorylation following inoculation with *F. mosseae* would increase ATP production, which is conducive to strong plant growth. Regarding the up-regulated amino acid metabolism, alanine, aspartate and glutamate pathways were particularly enriched in the treatment group (Figure 7B).

### Cluster Analysis of DEPs

**FIGURE 8:**
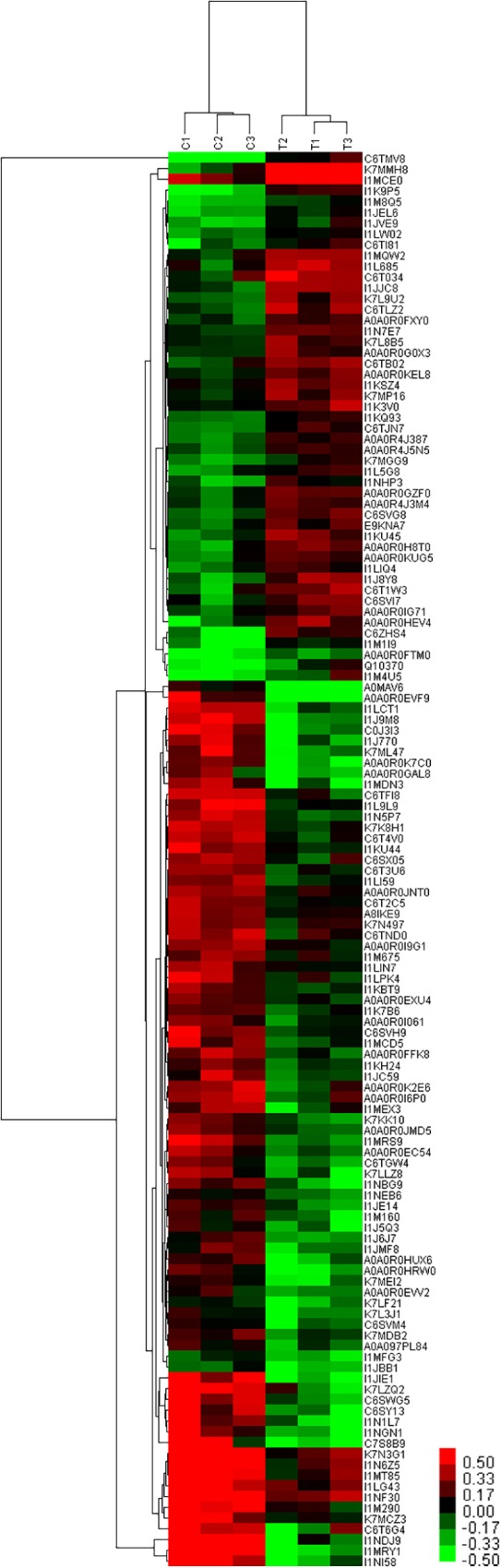
Cluster analysis of DEPs at 60 days after planting in continuous cropping for 1 year. C, control group; T, treatment group. 1, 2, and 3, parallel samples.

The results of cluster analysis are shown in Figure 8. Expression patterns of C1, C2, and C3 control groups, and T1, T2, and T3 treatment groups were the same. Significant differences were apparent between T and C groups. In total, 49 and 82 proteins were up- and down-regulated the T group. Protein clustering can provide subsets of proteins from the identified DEPs. Proteins with similar expression patterns may have similar functions or participate in the same biological pathway, or operate in adjacent regulatory positions in the pathway. When the positions were adjacent in clustering analysis, chalcone isomerase 4B (A0A0R4J387), pyruvate decarboxylase isozyme 2-like (A0A0R4J3M4), cytoplasmic-like isoform X1 (I1KQ93), NADH dehydrogenase subunit 6, and calreticulin-3-partial (K7L9U2) were up-regulated. These proteins are involved in defense systems and glucose metabolism, and may therefore promote plant sugar absorption and defense enzyme synthesis, and hence disease resistance. Expression of V-type proton ATPase subunit d2-like (A0A0R0IG71) was also up-regulated. V-type proton ATPases are primarily found in eukaryotes, and they function as proton pumps that acidify intracellular compartments and, in some cases, transport protons across the plasma membrane (PM). Some studies have demonstrated that mycorrhizal infection regulates the H+-ATP activity of the PM (Bago et al., 1997; Benabdellah et al., 1999). Meanwhile, Ferrol et al. (2002) demonstrated that mycorrhizal infection could up-regulate H+-ATP activity in wild-type tomato leaves, but not in mutant strains lacking mycorrhizal infection. The authors concluded that mycorrhizal regulation of H+-ATP activity could also be due to signal transduction pathways that may trigger and regulate plant responses to stress (Ferrol et al., 2002).

### Phenylalanine Ammonia-Lyase Activity

Metabolites produced by phenylpropane metabolic pathways play a very important role in plant disease resistance, of which flavonoid compounds are particularly important. Key enzymes in this pathway include phenylalanine ammonia lyase and chalcone synthase.

**FIGURE 9:**
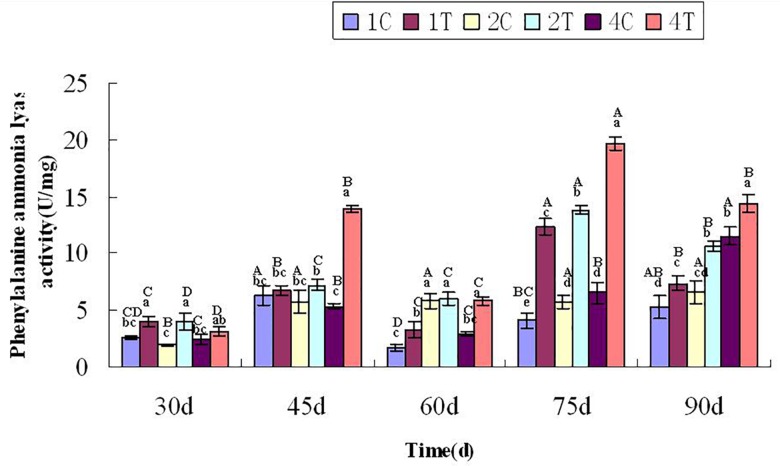
Phenylalanine ammonia-lyase activity of continuously cropped soybean roots at different growth stages. C, control group; T, treatment group. 1, 2, and 4 represents the years of continuous cropping. The small letters denote the significant differences among the different treatments at the same growth stages; the capital letters denote the significant differences among the same treatment at different growth stages. The differences in each parameter were detected at *P* < 0.05 level.

At 30 days after sowing, root samples from different treatments were randomly selected every 15 days. For each sampling period, the control group was compared with the treatment group. After thestatistical analysis, the treatment group displayed extremely significant differences compared with the control group (*P* < 0.05). Inoculation with *F. mosseae* generally increased the activities of defensive enzymes in soybean roots, which reduced the incidence of root rot and alleviated some of the problems associated with continuous cropping (Figure 9). And with the growth of the soybean, the phenylalanine ammonia-lyase activity increased significantly (*P* < 0.05). The phenylalanine ammonia-lyase activity was significantly affected by the sampling time, treatment and their interactions (*P* < 0.05; Table1).

## Discussion

Proteins act together to carry out the myriad of physiological functions taking place in plants and other organisms. To understand the impact of *F. mosseae* on biological systems in soybean suffering root rot, we must explore the structures and functions of root proteins, and changes that occur following inoculation with *F. mosseae* to clarify the regulatory mechanisms of this AMF under pathological conditions.

The potential of microbial symbiosis to prime the immune system of plant for pathogen attack has received more and more attention. Desalegn investigated the effect of beneficial microbes on the *Pisum* leaf proteome and metabolome as well as phenotype characteristics of plants in various symbiont interactions (mycorrhiza, rhizobia, co-inoculation, non-symbiotic) after infestation by *Didymella pinodes*. In healthy plants, mycorrhiza and rhizobia induced changes in RNA metabolism and protein synthesis (Desalegn et al., 2016). Song studied that AM can colonize the roots of *Amorpha fruticosa*, and have significant promoting effects on *A. fruticosa* growth as the intensity of fungal colonization increases (Song et al., 2015). Recorbet et al. (2010) have compared the root proteome responses of *Medicago truncatula* upon colonization with two AM fungi. Wang (2013) studied the dynamic changes in maize leaf protein expression profiles under AMF colonization. AMF play a significant role in promoting the growth of host plants, and researchers have increased their efforts to study the interactions between AMF and host plants.

The analysis of the data relating to the genes and proteins in AM symbiosis is important. Kong has found 47 symbiosis-related unigenes during AMF treatment. Among the expressed genes, those related to plant metabolism and stress and defense show important roles during the symbiotic process of AMF- *A. fruticosa* (Song et al., 2014). In AM roots, the PM of the host plant is involved in all developmental stages of the symbiotic interaction, from initial recognition to intracellular accommodation of intra-radical hyphae and arbuscules. Aloui performed a comparative protein profiling of PM fractions from *M. truncatula* roots either inoculated or not with the AM fungus Rhizophagus irregularis. This workflow identified a set of 82 mycorrhiza-responsive proteins that provided insights into the plant PM response to mycorrhizal symbiosis (Aloui et al., 2017).

### Specificity of Protein Alignment

The results of KEGG analysis revealed the up-regulation of three proteins aligned in two metabolic pathways (bile secretion and African trypanosomiasis), neither of which exist in plants. This may be due to the fact that KEGG analysis does not distinguish species when assessing protein enrichment, and there are few studies on these proteins. This form of computational analysis compares homologous proteins, which happen to be non-plant proteins in this case, hence metabolic pathways not present in plants can appear in the results of KEGG. Thus, improvements are needed in terms of species specificity for protein alignment when performing this analysis. Follow-up related experiments mapped the proteins to *Glycine max* proteins.

### Metabolism-Related Proteins

*Funneliformis mosseae* significantly promotes the growth and development of soybean plants, enhances the metabolic functions of plants in general, and provides a material and energy basis for the production of soybeans. The main metabolic processes in which the identified DEPs are involved include sugar metabolism, energy metabolism, amino acid metabolism, nucleotide anabolism, and lipid metabolism.

Glucose metabolism plays an important role in the growth and development of plants. In the present study, glucan endo-1,3-beta-glucosidase, pyruvate decarboxylase isozyme, sugar transport 5-like and cytoplasmic-like isoform X1 were up-regulated, potentially promoting glycolysis, gluconeogenesis, the biosynthesis and metabolism of the phosphopentose branch, as well as hexose, galactose and sucrose in soybean roots, consistent with a previous report (Wu et al., 2017).

Energy metabolism is one of the basic metabolic pathways in plant, and AMF can promote photosynthesis and respiration in plants (Porcel et al., 2015; Romero-Munar et al., 2017). Stress inhibits photosynthesis and respiration in plants, and energy loss inhibits plant growth and accelerates cell death. Energy storage promotes carbohydrate metabolism, providing energy for other metabolic pathways. In our experiments, expression of NADH dehydrogenase subunit 6 was up-regulated mitochondria, as was mitochondrial electron transport chain complex I that promotes the transfer of electrons from NADH to ubiquinone, as well as NADP metabolism and regeneration of NADPH. This indicates that *F. mosseae* likely accelerates oxidative phosphorylation and enhances the efficiency of the respiratory pathway in plants, thereby promoting the generation of energy.

### Defense-Related Proteins

Glucan 1,3-beta-glucosidase, an important disease-associated enzyme belonging to the PR2 family, is involved in the physiological development of cell division, fruit maturation and seed germination in plants. It is also involved in the composition of cell walls in many pathogenic fungi (Balasubramanian et al., 2012). In our experiments, glucan 1,3-beta-glucosidase was up-regulated, which may aid resistance to of root rot pathogens, indicating that *F. mosseae* likely induces disease defense responses in soybean, consistent with a previous report (Ramada et al., 2016).

Chalcone isomerase is involved in the biosynthesis of various defensive products in the phenylalanine metabolic pathway in plants, and plays an important role in antibacterial mechanisms, resistance to stress, cell development and differentiation, pigment accumulation and exogenous gene expression (Zorenc et al., 2017). This key enzyme in the flavonoid metabolic pathway is located downstream of chalcone synthase, and is essential for flavonoid compound production in plants (Park et al., 2018). In the treatment group in the present work, expression of chalcone isomerase 4B was up-regulated, which promotes the production of flavonoids, indicating that *F. mosseae* may induce defense responses in soybean, and thereby aid disease resistance.

The annexin family, also known as Ca^2+^-dependent phospholipid-binding proteins, are a class of homogenous, water-soluble, multifunctional proteins found in plants, animals and unicellular organisms that play an important role in plant growth and development, and the response to environmental stress (Wang et al., 2018; Zhou et al., 2018). Annexin genes are differentially expressed in various tissues in rape flowers under different hormone and stress conditions (Yadav et al., 2015). The ANN8 annexin gene in *Arabidopsis thaliana* is associated with disease resistance, and is expressed in the cytoplasm of specific plant cells. Expression levels are high in leaves, and is significantly increased under the induction of powdery mildew and *Pseudomonas syringae* (Lei and Wang, 2014). In our experiments, expression of annexin RJ4 was up-regulated, indicating that *F. mosseae* may induce stress responses and help in the resistance to invasion by pathogenic bacteria.

In conclusion, inoculation with *F. mosseae* promotes the expression of various disease resistance-associated, metabolic and energy-related proteins in soybean roots, thereby enhancing disease resistance and promoting plant growth and development.

## Conclusion

In this study, iTRAQ labeling and LC-MS/MS were employed to identify DEPs in continuously cropped soybean roots inoculated with *F. mosseae*. Of the 131 DEPs identified, many are involved in oxidative phosphorylation, glycolysis, amino acid metabolism, and other metabolic pathways. Glucan 1,3-beta-glucosidase, chalcone isomerase, calcium-dependent phospholipid-binding protein, and other defense-related proteins were up-regulated by *F. mosseae*. The findings provide an experimental basis for further research on the molecular mechanisms of AMF in resolving problems associated with continuous soybean cropping, and provide a theoretical and practical basis for the application of AMF as biological control agents to prevent soybean root rot.

## Availability of Data and Material

The datasets supporting the conclusions of this article are included within the article. The mass spectrometry proteomics data of this article are available in the ProteomeXchange Consortium and the data set identifier is pxD009911.

## Author Contributions

LB conceived, designed, and performed the experiments and polished the manuscript. H-BS analyzed the data. R-TL drew the diagrams. B-YC wrote the paper. All authors read and approved the final manuscript.

## Conflict of Interest Statement

The authors declare that the research was conducted in the absence of any commercial or financial relationships that could be construed as a potential conflict of interest.

## Footnotes

1 http://www.matrixscience.com

2 http://www.geneontology.org

3 http://www.kegg.jp/kegg/

## References

[B1] Abdallah C. Valot B. Guillier C. Mounier A. Balliau T. Zivy M. (2014). The membrane proteome of *Medicago truncatula* roots displays qualitative and quantitative changes in response to arbuscular mycorrhizal symbiosis. *J. Proteom.* 108 354–368. 10.1016/j.jprot.2014.05.028 24925269

[B2] Alojz K. Iztok A. Marta D. Mateja P. Matevž L. Katarina V. (2017). Arbuscularmycorrhizal fungi alter Hg root uptake and ligand environment as studied by X-ray absorption fine structure. *Environ. Exp. Bot.* 133 12–23. 10.1016/j.envexpbot.2016.09.006

[B3] Aloui A. Recorbet G. LemaîtreGuillier C. Mounier A. Balliau T. Zivy M. (2017). The plasma membrane proteome of *Medicago truncatula* roots as modified by arbuscular mycorrhizal symbiosis. *Mycorrhiza* 28 1–16. 10.1007/s00572-017-0789-5 28725961

[B4] Alva B. M. Zapata J. R. Roberts L. A. Tabla V. P. (2017). Effects of arbuscular mycorrhizal fungi on above-ground tri-trophic interactions are contingent upon plant genetic effects of cross type in the perennial herb *Ruellia nudiflora*. *J. Ecol.* 106 1133–1141. 10.1111/1365-2745.12859

[B5] Applied Biosystems (2004). iTRAQ^TM^ reagents amine-modifying labeling reagents for multiplexed relative and absolute protein. *Appl. Biosyst.* Available at: http://docs.appliedbiosystems.com/pebiodocs.04350831.pdf

[B6] Bago B. Donaire J. P. Azconaguilar C. (1997). ATPase activities of root microsomes from mycorrhizal sunflower (*Helianthus annuus*) and onion (*Allium cepa*) plants. *New Phytol.* 136 305–311. 10.1046/j.1469-8137.1997.00741.x

[B7] Balasubramanian V. Vashisht D. Cletus J. Sakthivel N. (2012). Plant β-1,3-glucanases: their biological functions and transgenic expression against phytopathogenic fungi. *Biotechnol. Lett.* 34:1983. 10.1007/s10529-012-1012-6 22850791

[B8] Benabdellah K. Azcónaguilar C. Ferrol N. (1999). Plasma membrane ATPase and H+ transport activities in microsomal membranes from mycorrhizal tomato roots. *J. Exp. Bot.* 50 1343–1349. 10.1093/jxb/50.337.1343

[B9] Bona E. Marsano F. Massa N. Cattaneo C. Cesaro P. Argese E. (2011). Proteomic analysis as a tool for investigating arsenic stress in *Pteris vittata* roots colonized or not by arbuscular mycorrhizal symbiosis. *J. Proteom.* 74 1338–1350. 10.1016/j.jprot.2011.03.027 21457805

[B10] Desalegn G. Turetschek R. Kaul H. P. Wienkoop S. (2016). Microbial symbionts affect pisum sativum, proteome and metabolome under *Didymella pinodes*, infection. *J. Proteom.* 143 173–187. 10.1016/j.jprot.2016.03.018 27016040

[B11] Fauziyah N. Hadisutrisno B. Suryanti S. (2017). The roles of arbuscular mycorrhizal fungi in the intensity of the foot rot disease on pepper plant from the infected soil. *J. Degrad. Mining Lands Manage.* 4 937–943. 10.15243/jdmlm.2017.044.937

[B12] Ferrol N. Pozo M. J. Antelo M. Azcón-Aguilar C. (2002). Arbuscular mycorrhizal symbiosis regulates plasma membrane H+-ATPase gene expression in tomato plants. *J. Exp. Bot.* 53 1683–1687. 10.1093/jxb/erf014 12096108

[B13] Gao S. Yan R. Cao M. Yang W. Wang S. Chen F. (2008). Effects of copper on growth, antioxidant enzymes and phenylalanine ammonia-lyase activities in *Jatropha curcas* L. seedling. *Plant Soil Environ.* 54 117–122. 10.17221/2688-PSE

[B14] Jung S. Rickert D. A. Deak N. A. Aldin E. D. (2003). Comparison of kjeldahl and dumas methods for determining protein contents of soybean products. *J. Am. Oil Chem. Soc.* 80:1169 10.1007/s11746-003-0837-3

[B15] Lei Y. Wang W. (2014). *The Annexin Gene ANN8 Negatively Regulates RPW8.1-Mediated Cell Death and Disease Resistance in Arabidopsis.* Dissertation, Sichuan Agricultural University, Ya’an.

[B16] Li L. Wei L. Cui J. Cai B. (2016). Effects of *Funneliformis mosseae* on the arbuscular mycorrhizal fungal community structure of root systems at the branching stage of continuous soybean cropping. *Mycosystema* 35 882–891.

[B17] Li N. Chen J. Yang F. F. Wei S. T. Kong L. G. Ding X. H. (2017). Identification of two novel *Rhizoctonia solani*-inducible cis-acting elements in the promoter of the maize gene. GRMZM2G315431. *Sci. Rep.* 7:42059. 10.1038/srep42059 28163300 PMC5292686

[B18] Lin Y. Zou W. Lin S. Onofua D. Yang Z. Chen H. (2017). Transcriptome profiling and digital gene expression analysis of sweet potato for the identification of putative genes involved in the defense response against *Fusarium oxysporum* f. sp. *batatas*. *PLoS One* 12:e0187838. 10.1371/journal.pone.0187838 29131830 PMC5683638

[B19] Luginbuehl L. H. Menard G. N. Kurup S. Van-Erp H. Radhakrishnan G. V. Breakspear A. (2017). Fatty acids in arbuscular mycorrhizal fungi are synthesized by the host plant. *Science* 356 1175–1178. 10.1126/science.aan0081 28596311

[B20] Park S. H. Lee C. W. Cho S. M. Lee H. Park H. Lee J. (2018). Crystal structure and enzymatic properties of chalcone isomerase from the Antarctic vascular plant *Deschampsia antarctica* Desv. *PLoS One* 13:e0192415. 10.1371/journal.pone.0192415 29394293 PMC5796730

[B21] Porcel R. Redondo-Gómez S. Mateos-Naranjo E. Aroca R. Garcia R. Ruiz-Lozano J. M. (2015). Arbuscular mycorrhizal symbiosis ameliorates the optimum quantum yield of photosystem II and reduces non-photochemical quenching in rice plants subjected to salt stress. *J. Plant Physiol.* 185 75–83. 10.1016/j.jplph.2015.07.006 26291919

[B22] Puglisi I. De-Patrizio A. Schena L. Jung T. Evoli M. Pane A. (2017). Two previously unknown Phytophthora species associated with brown rot of Pomelo (*Citrus grandis*) fruits in Vietnam. *PLoS One* 12:e0172085. 10.1371/journal.pone.0172085 28208159 PMC5313238

[B23] Qi P. F. Balcerzak M. Rocheleau H. Leung W. We Y.-M. Ouellet T. (2016). Jasmonic acid an abscisic acid play important roles in hostepathogen interaction between *Fusariu graminearum* and wheat during the early stages of fusarium head blight. *Physiol Mol. Plant Pathol.* 93 39–48. 10.1016/j.pmpp.2015.12.004

[B24] Qian L. Yu W. J. Cui J. Q. Jie W. G. Cai B. Y. (2015). *Funneliformis mosseae* affects the root rot pathogen *Fusarium oxysporum* in soybeans. *Acta Agric. Scand. Sect. B Soil Plant Sci.* 65 320–327.

[B25] Ramada M. H. Steindorff A. S. Jr. Bloch C. Ulhoa C. J. (2016). Secretome analysis of the mycoparasitic fungus *Trichoderma harzianum* ALL 42 cultivated in different media supplemented with *Fusarium solani* cell wall or glucose. *Proteomics* 16 477–490. 10.1002/pmic.201400546 26631988

[B26] Recorbet G. Valot B. Robert F. Gianinazzi-Pearson V. Dumas-Gaudot E. (2010). Identification of in planta-expressed arbuscular mycorrhizal fungal proteins upon comparison of the root proteomes of *Medicago truncatula* colonised with two glomus species. *Fungal Genet. Biol.* 47 608–618. 10.1016/j.fgb.2010.03.003 20226871

[B27] Romero-Munar A. Del-Saz N. F. Ribas-Carbó M. Flexas J. Baraza E. Florez-Sarasa I. (2017). Arbuscular mycorrhizal symbiosis with *Arundo donax* decreases root respiration and increases both photosynthesis and plant biomass accumulation. *Plant Cell Environ.* 40 1115–1126. 10.1111/pce.12902 28060998

[B28] Sharma I. P. Sharma A. K. (2017). Co-inoculation of tomato with an arbuscular mycorrhizal fungus improves plant immunity and reduces root-knot nematode infection. *Rhizosphere* 4 25–28. 10.1016/j.rhisph.2017.05.008

[B29] Shi Z. Y. Wang Y. M. Xu S. X. Lan Z. J. Mickan B. S. Zhang X. L. (2017). Arbuscular Mycorrhizal fungi enhance plant diversity, density and productivity of spring ephemeral community in desert ecosystem. *Notulae Botanicae Horti Agrobotanici Cluj-Napoca* 45 301–307. 10.15835/nbha45110766

[B30] Song F. Qi D. Liu X. Kong X. Gao Y. Zhou Z. (2015). Proteomic analysis of symbiotic proteins ofglomus mosseaeandamorpha fruticosa. *Sci. Rep.* 5:18031. 10.1038/srep18031 26658758 PMC4674871

[B31] Song F. Q. Kong X. S. Li J. Z. Chang W. (2014). Screening the related genes in the AM fungi and symbiosis with the suppression subtractive hybridization technique. *Sci. Silvae Sin.* 11 64–74.

[B32] Tao L. L. Ahmad A. de-Roode J. C. Hunter M. D. (2016). Arbuscular mycorrhizal fungi affect plant tolerance and chemical defences to herbivory through different mechanisms. *J. Ecol.* 104 561–571. 10.1111/1365-2745.12535

[B33] Thermo Fisher Scientific Inc. (2012). *Proteome Discoverer Version 1.4.* Waltham, MA: Thermo Fisher Scientific.

[B34] Tripathi S. Mishra S. K. Varma A. (2017). “Mycorrhizal fungi as control agents against plant pathogens,” in *Mycorrhiza-Nutrient Uptake, Biocontrol, Ecorestoration*, eds Varma A. Prasad R. Tuteja N. (Berlin: Springer).

[B35] Wan Y. Bureau Q. (2011). Influence of soybean moisture content and smash degree on crude fat content tested by Soxhlet extraction. *Port Health Control* 2 35–37.

[B36] Wang R. Mei Y. Xu L. Zhu X. Wang Y. Guo J. (2018). Differential proteomic analysis reveals sequential heat stress-responsive regulatory network in radish (*Raphanus sativus* L.) taproot. *Planta* 247 1432–2048. 10.1007/s00425-018-2846-5 29368016

[B37] Wang Z. (2013). Identification and functional analysis of maize leaf proteins responding to the abuscular mycorrhizal fungi (amf). *Chin. J. Trop. Agric.* 33 40–44.

[B38] Wei M. Zhu J. Q. Guan W. X. Zhang W. B. Fu Z. Wang L. H. (2017). First report of *Fusarium avenaceum* causing root rot of maca (*Lepidium meyenii*) in China. *Plant Dis.* 101:832 10.1094/PDIS-07-16-1061-PDN

[B39] Wu H. H. Zou Y. N. Rahman M. M. Ni Q. D. Wu Q. S. (2017). Mycorrhizas alter sucrose and proline metabolism in trifoliate orange exposed to drought stress. *Sci. Rep.* 7:42389. 10.1038/srep42389 28181575 PMC5299426

[B40] Xu X. H. Chen C. Zhou Z. Sun Z. H. Chen Y. H. Jiang J. D. (2017). The influence of environmental factors on communities of arbuscular mycorrhizal fungi associated with *Chenopodium ambrosioides* revealed by MiSeq sequencing investigation. *Sci. Rep.* 7:45134. 10.1038/srep45134 28327631 PMC5361092

[B41] Yadav D. Ahmed I. Kirti P. B. (2015). Genome-wide identification and expression profiling of annexins in *Brassica rapa*, and their phylogenetic sequence comparison with *B. juncea*, and *A. thaliana*, annexins. *Plant Gene.* 4 109–124. 10.1016/j.plgene.2015.10.001

[B42] Zhou A. Sun H. Feng S. Zhou M. Gong S. Wang J. (2018). A novel cold-regulated gene from Phlox subulata, PsCor413im1, enhances low temperature tolerance in *Arabidopsis*. *Biochem. Biophys. Res. Commun.* 495 1688–1694. 10.1016/j.bbrc.2017.12.042 29229392

[B43] Zhu Y. Xu H. Chen H. Xie J. Shi M. Shen B. (2014). Proteomic analysis of solid pseudopapillary tumor of the pancreas reveals dysfunction of the endoplasmic reticulum protein processing pathway. *Mol. Cell. Proteom.* 13 2593–2603. 10.1074/mcp.M114.038786 24997997 PMC4188989

[B44] Zorenc Z. Veberic R. Slatnar A. Koron D. Miosic S. Chen M. H. (2017). A wild ’albino’ bilberry (*Vaccinium myrtillus* L.) from Slovenia shows three bottlenecks in the anthocyanin pathway and significant differences in the expression of several regulatory genes compared to the common blue berry type. *PLoS One* 12:e0190246. 10.1371/journal.pone.0190246 29272302 PMC5741254

